# Iron Is a Sensitive Biomarker for Inflammation in Multiple Sclerosis Lesions

**DOI:** 10.1371/journal.pone.0057573

**Published:** 2013-03-14

**Authors:** Veela Mehta, Wei Pei, Grant Yang, Suyang Li, Eashwar Swamy, Aaron Boster, Petra Schmalbrock, David Pitt

**Affiliations:** 1 Department of Neurology, The Ohio State University, Columbus, Ohio, United States of America; 2 Department of Radiology, The Ohio State University, Columbus, Ohio, United States of America; 3 Department of Neurology, School of Medicine, Yale University, New Haven, Connecticut, United States of America; Innsbruck Medical University, Austria

## Abstract

MRI phase imaging in multiple sclerosis (MS) patients and in autopsy tissue have demonstrated the presence of iron depositions in white matter lesions.

The accumulation of iron in some but not all lesions suggests a specific, potentially disease-relevant process, however; its pathophysiological significance remains unknown.

Here, we explore the role of lesional iron in multiple sclerosis using multiple approaches: immunohistochemical examination of autoptic MS tissue, an *in vitro* model of iron-uptake in human cultured macrophages and ultra-highfield phase imaging of highly active and of secondary progressive MS patients.

Using Perls' stain and immunohistochemistry, iron was detected in MS tissue sections predominantly in non-phagocytosing macrophages/microglia at the edge of established, demyelinated lesions. Moreover, iron-containing macrophages but not myelin-laden macrophages expressed markers of proinflammatory (M1) polarization.

Similarly, in human macrophage cultures, iron was preferentially taken up by non-phagocytosing, M1-polarized macrophages and induced M1 (super) polarization. Iron uptake was minimal in myelin-laden macrophages and active myelin phagocytosis led to depletion of intracellular iron.

Finally, we demonstrated in MS patients using GRE phase imaging with ultra-highfield MRI that phase hypointense lesions were significantly more prevalent in patients with active relapsing than with secondary progressive MS.

Taken together, our data provide a basis to interpret iron-sensitive GRE phase imaging in MS patients: iron is present in non-phagocytosing, M1-polarized microglia/macrophages at the rim of chronic active white matter demyelinating lesions. Phase imaging may therefore visualize specific, chronic proinflammatory activity in established MS lesions and thus provide important clinical information on disease status and treatment efficacy in MS patients.

## Introduction

Multiple sclerosis (MS) is an inflammatory disease of the central nervous system (CNS) characterized by infiltration of immune cells and subsequent loss of myelin, oligodendrocytes, and axons [1]. Conventional magnetic resonance imaging (MRI) is used routinely for diagnosis and for monitoring of disease activity. Inflammation is detected using gadolinium-diethylene-triamine-penta-acetic-acid, which visualizes breakdown of the blood–brain barrier (BBB). However, experimental imaging studies in MS patients that trace activated microglia with PET imaging [2] or detect infiltrating monocytes with ultra-small iron oxide particle enhancement [3] visualize patterns of inflammation distinct from Gd-enhancement. Thus, inflammation can occur in the context of an intact BBB and additional imaging modalities are required to obtain a more complete picture of the inflammatory activity in MS patients.

Gradient-echo (GRE) phase imaging at ultra-highfield MRI is highly sensitive for iron. A number of GRE studies with MS patients and autoptic MS tissue, including our own [4], [5], [6], [7], [8], have demonstrated that iron accumulates in white matter and cortical lesions. Several patterns of phase signal within lesions have been identified, including nodular lesions that were uniformly phase hypointense, lesions with a hypointense rim at the margin and lesions containing veins [4], [5], [7], [9]. In a recent study that combined ultra-highfield phase MR imaging with histopathological analysis, Bagnato and colleagues correlated MRI phase signal with presence of iron within histological sections [7]. The authors confirmed that phase imaging detects iron in brain tissue with high sensitivity and identified the cellular localizations of iron depositions. In white matter MS lesions, iron was present within microglia/macrophages at the lesion perimeter. In contrast, in normal appearing white matter, iron was found in oligodendrocytes, confirming prior reports that oligodendrocytes are the major iron-containing cells in the adult CNS. In addition, iron precipitates were present in hemosiderin aggregates within and outside of white matter MS lesions suggestive of remote microhemorrhages. Thus, while iron is not specific to one cell type or to one pathological process, the topographical context provided by MR images allows distinguishing the different sources of iron within the MS brain. Particularly, iron deposition at the lesion rim is likely to localize to activated microglia/macrophages.

The presence of iron deposition in microglia/macrophages in some but not all lesions suggests a specific process; however, the functional significance of iron-rich microglia/macrophages for lesion development is unknown. Moreover, the prevalence of lesional iron in different stages of MS has not been examined. To investigate these questions, we used several complementary approaches: histological examination of autopsied MS tissue, iron-uptake studies in human monocyte-derived macrophage cultures and ultra-highfield (7T) MR imaging of MS patients.

Our findings suggest that iron-sensitive phase imaging detects pro-inflammatory M1 activity and may thus provide clinically relevant information on the inflammatory status of MS lesions.

## Methods

### Iron-sensitive imaging in MS patients

8 MS patients with active relapsing-remitting MS and 8 patients with secondary progressive MS were scanned (see table 1). For the purpose of this study, active MS was defined as having one or more relapses and/or one or more Gd-enhancing lesions within the last 9 months. Secondary progressive MS was defined as having had no relapse for at least 6 years and no radiological evidence of new lesion formation (no Gd-enhancing lesions and stable T2 lesion load as compared to a previous MRI≥1 years). The average EDSS score was 2.9 for RR-MS patients and 4.2 for SP-MS patients.

**Table 1:** Patient demographics.

	Clinical data					3T/7T imaging	
	MS type	Age	Disease duration [years]	Last clinical relapse prior to 7T scan [years]	Last Gd^+^MRI prior to 7T scan [years]	iron^+^leisions [n]	iron^+^/FLAIR [%]
Pt 1	RR-MS	37	0.2	0.1	present scan	15	26.9
Pt 2	RR-MS	30	7	1.1	0.5	17	25.4
Pt 3	RR-MS	33	3	3	0.1	16	28.8
Pt 4	RR-MS	31	4	10	present scan	15	25.5
Pt 5	RR-MS	28	4	0.7	none documented	2	20.2
Pt 6	RR-MS	30	4	0.3	presnet scan	20	51.7
Pt 7	RR-MS	45	7	0.2	none documented	11	64.3
Pt 8	RR-MS	53	4	0.6	none documented	5	27
Pt 9	RR-MS	55	25	20	none documented	0	0
Pt 10	RR-MS	54	22	15	10	2	4.9
Pt 11	RR-MS	58	13	11	13	1	3.2
Pt 12	RR-MS	62	36	16	14	1	7.7
Pt 13	RR-MS	66	33	28	none documented	3	9.5
Pt 14	RR-MS	45	17	17	none documented	6	15
Pt 15	RR-MS	56	18	16	none documented	0	0
Pt 16	RR-MS	65	10	7	3	2	14.3

The patients had varying treatment histories and were on different disease-modifying treatments at the time of the study. Two patients with secondary progressive MS were on no disease-modifying treatment.

Patients were scanned on both a whole-body 7T MRI (Achieva; Philips Medical Systems, Best, The Netherlands) equipped with a 16-channel receive phased array coil with a head transmitter coil (Nova Medical Inc.) and on a 3T MRI (Achieva, Philips Medical Systems equipped with a 8-channel head coil). At 7T, volumetric 3D-GRE sequences were acquired with a TR/flip angle of 30 ms/5°, 4–5 echoes ranging from TE = 5 to 25 ms and 0.5×0.5×1.0 mm^3^ acquired voxel size. At 3T, T2-FLAIR and pre and post Gd contrast T1-weighted images were acquired using conventional clinical protocols. To match 3T and 7T images, data for each subject were registered using FSL. Iron-sensitive images were generated from the complex 7T GRE images with high pass filtering.

In addition to conventional magnitude images, complex images were generated using scanner software which included combination of data from multiple receiver coils. Off-line processing of the saved complex images with in-house software included inverse Fourier transformation, multiplication with a Gaussian filter and subtraction of the filtered from the original k-space data [10].Fourier transform of the filtered data generated phase images devoid of spatially slowly varying phase banding artifacts.

White matter MS lesions were identified on 3T FLAIR/T2WI images on 4 predefined levels and matched with signal hypointensities on co-registered 7T phase images. Patients were recruited in the Ohio State University MS Center. Patient imaging was approved by the institutional review board.

### Tissue Samples

Human CNS tissue was obtained at autopsy according to an Institutional Review Board-approved protocol. CNS tissue was obtained from 6 subjects with MS and 2 subjects with non-neurological diseases. Postmortem intervals were between 4 and 12 hours. Brain tissue was fixed by immersing the brain in 10 to 20% formalin for 2 to 4 weeks. Some samples were embedded in paraffin; other samples were stored in formalin.

Patient 1 was a 32-year-old woman, 4-year disease duration, cause of death: sepsis; Patient 2 was a 42-year-old man, 6-year disease duration, cause of death: bronchopneumonia; Patient 3 was a 38-year-old woman, 11-year disease duration, cause of death: UTI/urosepsis and Patient 4 was a 31-year-old woman, 8-year disease duration, cause of death: respiratory failure. Two patients had SPMS and displayed chronic silent lesions only: Patient 5 was a 59-year-old woman, 32-year disease duration, cause of death: myocardial infarct, and Patient 6 was a 69-year-old man, 12-year disease duration, cause of death: bronchopneumonia. Non-MS CNS tissue was from 2 patients dying from non-neurologic causes (myocardial infarct and acute myeloblastic leukemia).

Only one patient was treated with disease-modifying treatments, including β-interferon and cellcept; the other patients died before disease-modifying treatments were widely available. The EDSS scores of the patients at the time of their deaths are not known.

### Histochemistry and Immunohistochemistry

For basic characterization, formalin-fixed sections were stained using hematoxylin and eosin and with Luxol fast blue myelin staining. Iron was detected with DAB-enhanced Perls' staining (Prussian blue reaction). For this, slides were immersed in 4% ferrocyanide/4% hydrochloric acid for 30 minutes in the dark. Iron staining was enhanced through incubation with 3,3-diaminobenzidine (Vector Laboratories) for 30 minutes at room temperature. Sections were rinsed with distilled water, dehydrated, and coverslipped with Permount (Vector Laboratories).

Myelin debris within macrophages was detected with oil red-O (ORO; Sudan Red 5B). ORO powder was dissolved in isopropanol at a concentration of 0.5% and further diluted at a 3∶5 dilution in dH_2_O. Sections were incubated with ORO for 10 minutes, washed with tap water and cover-slipped with aqueous mounting medium without dehydration.

Immunohistochemistry was performed on formalin-fixed sections that were quenched with 0.03% hydrogen peroxide and blocked with normal serum. Sections were then incubated with primary antibodies overnight and processed with the appropriate biotinylated secondary antibody and avidin/biotin staining kit with diaminobenzidene as chromogen (Vector ABC Elite Kit, Vector Laboratories).

Primary antibodies against the following antigens were used (table 2): myelin basic protein (myelin and oligodendrocytes), glial fibrillary acidic protein (astrocytes), CD68 (macrophages, microglia), ferritin, inducible nitric oxide synthase (a marker for classically activated macrophages), arginase-1 and mannose receptor [CD206] (both markers for alternatively activated macrophages). Negative controls included the use of irrelevant primary antibodies of the same isotype diluted to the same immunoglobulin concentration and omission of the primary antibody. When indicated, chromogenic signal was amplified by using Tyramide Signal Amplification (TSA) as per manufacturer's instructions (Perkin Elmer). Images were acquired with a BX41 Olympus microscope using MicroSuite Five imaging software.

**Table 2:** Antibodies.

Primary antibody	Dilution	Secondary antibody	Dilution
anti-human ferritin polyclonal(Abcam)	1∶600	biotynylated goat anti-rabbit IgG (Vector)	1∶500
anti-human CD68 monoclonal mouse IgG (Dako)	1∶300	biotynylated goat anti-mouse (Southern Biotech)	1∶300
anti-human CD206 polyclonal mouse IgG(R&D systems)	1∶10,000	biotynylated horse anti-goat IgG (Vector)	1∶1,000 w/TSA
anti-human iNOS polyclonal rabbit (Abcam)	1∶20,000	biotynylated goat anti-rabbit IgG (Vector)	1∶1,000 w/TSA
anti-human arginase-1 polyclonal goat (santaCruz)	1∶200	biotynylated horse anti-goat IgG (Vector)	1∶400
anti-human GFAP polyclonal chicken (Covance)	1∶2,000	biotynylated goat anti-chicken IgY (Vector)	1∶500
anti-human Myelin Basic Protein (MBP) polyclonal rabbit (Dako)	1∶3,000	biotynylated goat anti-rabbit IgG (Vector)	1∶500

### Lesion Classification

MS lesions were categorized into early active, chronic active and chronic silent lesions. Early active lesions were characterized by hypercellularity, edema, intense macrophage infiltration with ongoing demyelination, astroglial hypertrophy and absence of fibrous astrogliosis. Chronic-active lesions were characterized by central chronic demyelination and gliosis with inflammatory activity and possible active demyelination at the lesion margin. Chronic silent lesions consisted of areas of demyelination, astrogliosis and absence of inflammation.

### Human monocyte-derived macrophage (MDM) cell culture

Human peripheral blood monocytes (PBMC) were isolated from leucopacs obtained from the American Red Cross [11]. Monocytes were purified using positive selection with CD14-coated magnetic beads (Miltenyi Biotec). Alternatively, PBMCs (2×10^6^/ml) were cultured in Teflon wells (Savillex) for 6 days in RPMI1640 containing 10% FBS and 20 ng/ml M-CSF [12]. Adherent PBMCs containing macrophages were removed from Teflon wells, washed and plated in tissue culture plates. MDMs were further purified by adherence in tissue culture plates. Two hours after plating non adherent cells were removed by extensive washing with RPMI. For differentiation into macrophages (M0), purified monocytes were cultured in RPMI1640 supplemented with 10% FBS, and 20 ng/ml M-CSF for 6 days. Monocytes-derived macrophages (MDMs) were then polarized towards a M1 phenotype by incubation with LPS (1 µg/ml) and IFN-γ (100 U/ml) or a M2 phenotype with IL-4 (20 ng/ml) for three days [13]. FeCl_3_ was added at various concentrations (0, 25, 50, 100 µM) for 10 hrs before harvesting. In some experiments, human purified myelin (30 µg/ml) was added to macrophage cultures either alone or 24 hrs prior to addition of FeCl_3_. To determine cytokine concentrations released by macrophages, conditioned media were collected and centrifuged to remove cell debris. Supernatant was stored in −80°C until use. In selected experiments, fluorescently labeled polystyrene microspheres were added to macrophage cultures (diameter 1 µm; Bangs Laboratories) at a concentration of 1 µg/ml.

### Myelin isolation

For myelin purification from human white matter tissue, we followed the commonly used method of sequential enrichment by two discontinuous sucrose density gradient centrifugations [14]. Briefly, white matter was dounce-homogenized in isotonic sucrose (0.32 M) and layered over 0.85 M sucrose. Following centrifugation, myelin-enriched fraction was obtained at the interphase, washed twice with water and then purified further by second discontinuous sucrose gradient centrifugation. Sucrose was removed by additional three washes with water followed by single wash in phosphate buffered saline.

### Analysis of cellular ^55^Fe uptake

Non-polarized and M1 or M2 polarized macrophages cultured in 96 well plates were incubated with a mixture of ^55^FeCl_3_ (2 µCi/µM; PerkinElmer) and unlabeled FeCl_3_ for a final concentrations of 0, 25, 50 and 100 µM. Some wells received myelin (30 µg/ml) 24 h prior to addition of the radioactive iron. Cells were incubated for 14 hrs, washed and lysed in 1.0 M NaOH. ^55^Fe within the cellular lysates was measured by liquid scintillation counting.

### Measurement of oxidative burst in macrophages

Generation of reactive oxygen species (ROS) by macrophages in response to iron uptake were measured with the redox-sensitive cellular probe DCF-DA (Life Technology) as per manufacturer's instruction [15]. Briefly, monocyte-derived macrophages were pre-incubated with various concentrations of FeCl_3_ (0–100 µM) for 2 h prior to addition of 30 µM of DCF-DA. The kinetics of ROS generated by macrophages was captured using an Infinite 500 plate reader (Tecan) Addition of H_2_O_2_ (5 µM/ml) to macrophages for 30 minutes was used as a positive control. For visualization of ROS, macrophages grown on coverslips were incubated with 10 µM DCF-DA for 30 min, washed with PBS and mounted with aqueous mounting media (Thermo Fisher Scientific) on glass slides.

### Immunocytochemistry

Macrophages grown on coverslips were fixed with 4% paraformaldehyde/PBS (wt/vol) for 20 min at room temperature and permeabilized with 0.1% Triton X-100 for 15 min at room temperature. Cells were incubated overnight at 4°C with the anti-ferritin (1∶200) antibody followed by incubation with appropriate Alexa-flour 488 coupled secondary antibodies (Invitrogen). Slides were mounted with DAPI-containing aqueous mounting media.

### Enzyme-linked immunosorbent assays (ELISA)

Cytokine concentrations in cell supernatants (TNF-α, IL-10) were measured by using sandwich ELISAs. Horseradish peroxidase-conjugated antibodies were used as developing antibodies and detected by color development with Pierce ABTS substrate (Thermo Fisher Scientific) using an EMAX plate reader at 405 nm (Molecular Devices). Cytokine standards and samples were assayed in triplicate. Concentrations were calculated from standard curves generated for each cytokine.

### Statistics

Student t test was performed to compare non-phagocytosing and myelin-laden macrophages. The Mann-Whitney test was used to compare the lesion counts between inflammatory active and secondary progressive MS patients. All data were analyzed with GraphPad Prism software, version 6.0a. P-values less than 0.05 (two-tailed) were considered as statistically significant.

## Results

### Iron is present in non-phagocytosing, M1-polarized macrophages in chronic active MS lesions

**Figure 1:**
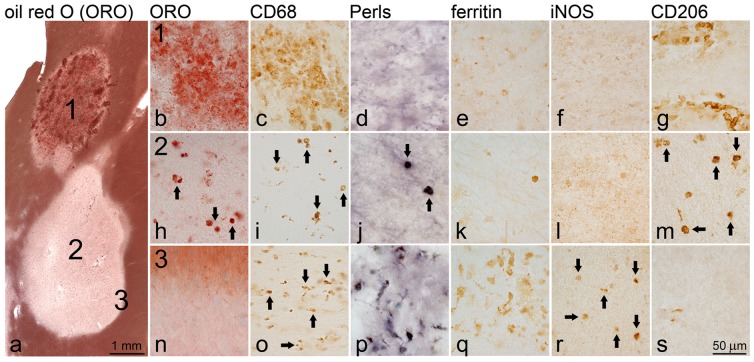
Iron deposition in white matter MS plaques. The overview image [a] shows an oil-red O-stained section with an actively demyelinating lesion [1] and a demyelinated lesion [2]. The actively demyelinating lesion (1) contains an abundance of large, myelin-laden macrophages as indicated by the presence of oil-red O positive material within CD68^+^ macrophages [b, c]. These macrophages do not contain iron as indicated by Perls' staining [d], or the iron storage protein ferritin [e] and express the M2 polarization marker CD206 [g] but not iNOS [f]. In the demyelinated lesion [2], at least two populations of macrophages were observed: in the lesion center [2], small round CD68^+^ macrophages contain condensed myelin [h, i]. Occasional macrophages contain iron [j] and correspondingly, ferritin [k] and express CD206 [m] but not iNOS [l]. In contrast, macrophages at the lesion rim (3) have a ramified appearance and do not contain oil-red O positive material [n, o]. These cells contain large amounts of iron [p] and iron-storing ferritin [q] and are iNOS positive [r] but CD206 negative [s], suggesting M1 polarization.

To investigate the cellular localization of iron in white matter plaques, we examined actively demyelinating, chronic active and chronic silent MS lesions (Fig. 1a). Myelin–laden (oil red-O [ORO]-positive) macrophages were present in high density in actively demyelinating lesions (Fig. 1a–c) and, in lower numbers, in the center of chronic active lesions (Fig. 1a, h, i; arrows). These cells contained no or small amounts of ferric iron (Fe^3+^) and the iron-storage protein ferritin whose expression is closely tied to intracellular iron content (Fig. 1d, e and j, k). Myelin-laden cells were typically strongly immunoreactive for CD206 (Fig. 1g, m) and arginase-1 (not shown) but not for iNOS (Fig. 1f, l), suggesting an anti-inflammatory polarization state.

In contrast, iron deposition was prominent in a subset of CD68-positive macrophages/microglia at the edge of chronic active lesions (Fig. 1a, o, p). These cells were also highly immunoreactive for ferritin, (Fig. 1q). Iron-positive macrophages were not myelin phagocytosing, based on their small size and ramified morphology, absence of intracellular oil red-O positive material (Fig. 1n, o) and absence of intracellular myelin basic protein (not shown). Moreover, macrophages/microglia at the lesion edge were immunoreactive for the classical activation (M1) marker iNOS (Fig. 1r) but not the alternative activation (M2) markers CD206 (Fig. 1s) and arginase-1, indicative of proinflammatory polarization of iron-positive cells.

Thus, two macrophage/microglia populations were identified in white matter MS lesions: (i) iron-positive, myelin-negative (i.e. non-phagocytosing), proinflammatory microglia/macrophages at the edge of chronic active lesions and (ii) iron-negative, myelin-laden macrophages with an anti-inflammatory phenotype present in actively demyelinating lesions and at the lesion center of demyelinated lesions.

Chronic silent white matter MS lesions contained no or only small amounts of iron (data not shown) while in cortical lesions, activated microglia at the lesion margins were invariably iron-positive, as reported before [6]. Other sources of iron included hemosiderin deposition in the vicinity of blood vessels within and outside of white matter MS lesions and iron-rich oligodendrocytes that were dispersed within normal appearing white matter (data not shown) consistent with prior reports [7], [16].

### Polarization and phagocytic activity determines iron accumulation in macrophages

**Figure 2:**
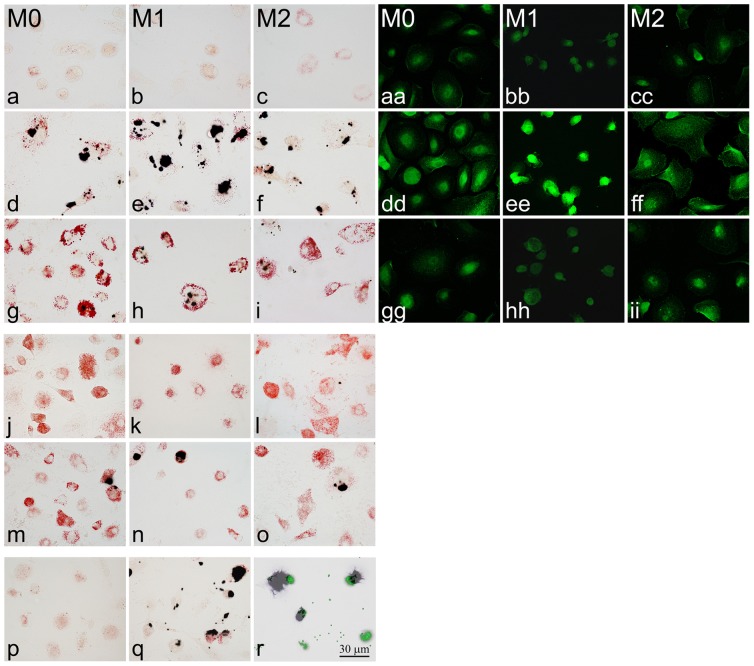
Iron and myelin uptake in human monocyte-derived macrophages (MDMs). Non-polarized M0, M1- and M2-polarized macrophages were stained for neutral lipids (oil red-O) and iron (Perls). In untreated cultures, macrophages did not contain iron or significant amounts of lipids [a–c]. In macrophages exposed to FeCl_3_ (10 µM; 10 hrs), Perls' staining showed polarization-dependent iron uptake [d–f]. Exposure of iron-rich macrophages to purified human myelin (30 µg/ml; 24 hrs) lead to depletion of iron from myelin-phagocytosing macrophages in all polarization states [g–i]. Presence of iron within macrophages was closely mirrored by macrophage expression of ferritin: macrophages without iron-load express little ferritin [aa–cc], while iron loading leads to increased expression of ferritin [dd–ff], particularly in M1 macrophages [ee]. Addition of myelin to iron-rich macrophages resulted in substantial reduction of ferritin in all polarization states [gg–ii]. In macrophages exposed first to myelin [j–l] and subsequently to iron, iron accumulation was significantly reduced compared to that in naive macrophages [m–o]. When M1 macrophages were incubated with myelin in the presence of the receptor-associated protein (GST-RAP), which binds to the presumed receptor for myelin ingestion, LRP-1, and inhibits interaction with other ligands, myelin ingestion was prevented [p]. Blockade of myelin ingestion in M1 macrophages with GST-RAP and subsequent exposure to FeCl_3_ led to unimpeded iron uptake [q]. In contrast to myelin, internalization of fluorescent polystyrene microspheres by M1 macrophages did not prevent subsequent iron uptake [r].

**Figure 3:**
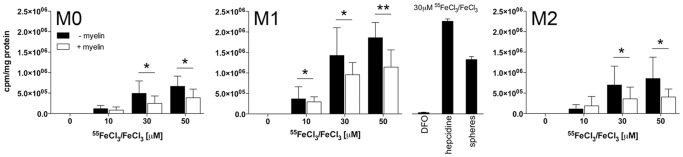
Myelin decreases ^55^Fe uptake by MDMs. Human MDMs were seeded in 96 well dishes at a cell density of 8×10^4^ cells/well and polarized as described in Methods. Incubation with increasing concentrations of ^55^FeCl_3_ and non-radioactive FeCl_3_ resulted in dose-dependent and polarization-dependent iron uptake (M1>M2>M0). When human myelin was added (30 µg/ml) 24 hrs prior to exposure with iron, iron uptake was significantly reduced in all polarization states. Adding fluorescent-labeled polystyrene microspheres (1 ug/ml) instead of myelin did not significantly reduce radioactive iron uptake. In separate experiments, the iron chelator desferoxamine (100 µM) and hepcidin, which prevents cellular iron export by binding to the iron channel ferroportin (700 nM), were added as controls. Cell-associated radioactivity was determined in triplicate samples. The mean value of three separate experiments (± SEM) are shown. Statistical significance was obtained by comparing the values between cells incubated with and without myelin. *p<0.05; ** p<0.01.

Next, we assessed how phenotypic polarization and myelin phagocytosis affects iron uptake in macrophages. We used established protocols to polarize M-CSF-elicited human macrophages towards M1 and M2 phenotypes. M0, M1 and M2 cells were exposed to varying concentrations of FeCl_3_ ranging from 0 to 100 µM. Iron uptake was visualized with Perls' stain in macrophages cultured on coverslips (Fig. 2) and in separate experiments quantified by addition of radioactive ^55^FeCl_3_ (Fig. 3). Iron uptake occurred in a dose dependent fashion and was most pronounced in M1-polarized macrophages shown with Perls' stain (Fig. 2a–f) and by cellular ^55^Fe liquid scintillation (Fig. 3).

Changes in cellular iron were mirrored by changes in expression of the iron storage protein ferritin (Fig. 2aa–ff). Iron accumulation was prevented in M1-polarized macrophages by co-incubation with the iron chelator, desferoxamine, and was enhanced with hepcidin, an iron regulatory hormone that sequesters iron in macrophages by binding to the iron export protein ferroportin (Fig. 3).

When iron-loaded macrophages were loaded with purified human myelin subsequent to iron uptake, iron content was substantially reduced in macrophages that contained myelin lipid droplets (Fig. 2g–i). Similarly, macrophages that were loaded with myelin prior to iron exposure (Fig. 2 j–l) incorporated a significantly smaller amount of iron than non-myelin laden macrophages (Fig. 2m–o, Fig. 3).

In contrast to myelin-laden macrophages, macrophages that were loaded with latex beads still maintained their ability to ingest iron, suggesting that blockade of iron uptake in myelin-positive macrophages might not be due to phagocytic overload but a more specific, possibly lipid-induced process (Fig. 2r and Fig. 3). To specifically block myelin ingestion, macrophages were incubated with glutathione-S-transferase receptor-associated protein (GST-RAP). GST-RAP binds to low-density lipoprotein receptor-related protein-1, an essential receptor for phagocytosis of myelin vesicles [17]. GST-RAP incubation resulted in blockade of myelin phagocytosis (Fig. 2p), which was associated with unimpeded iron uptake in macrophages (Fig. 2q).

### Iron uptake in macrophages promotes excessive M1 polarization

**Figure 4:**
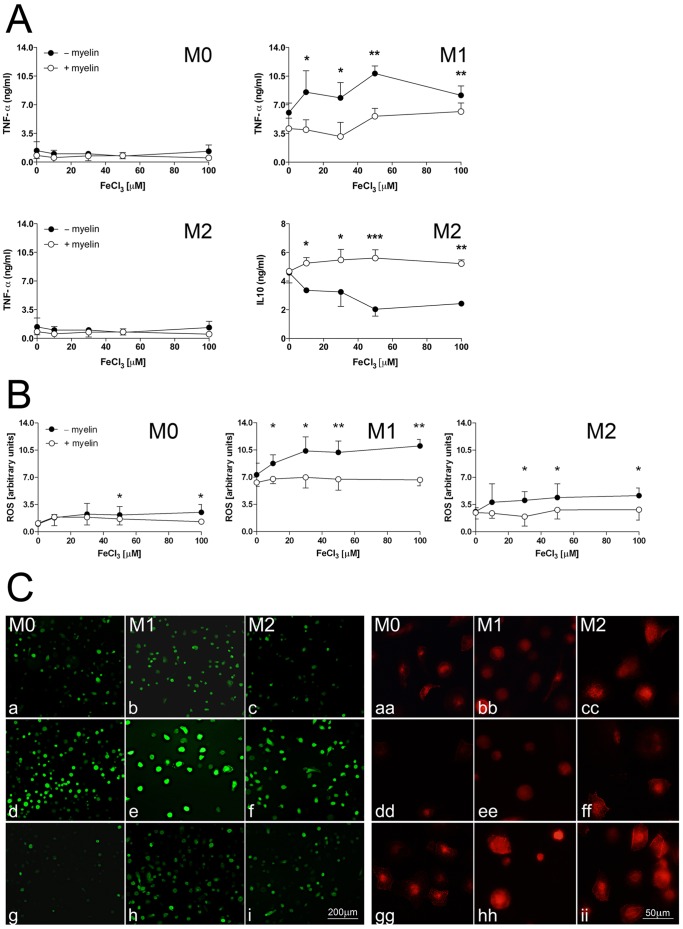
Iron uptake enhances M1 polarization. A. Human MDMs were seeded in 48 well dishes at cell density of 2×10^6^ cells/ml and polarized according to protocol. Various concentrations of FeCl_3_ (0–100 µM) were added to the differentially polarized macrophages. Where indicated, cells were pre-incubated with human myelin (30 µg/ml) for 24 hrs before addition of FeCl_3_. After 10 hrs, cell-free supernatants were collected and TNF-α and IL-10 concentrations were measured by ELISA. Iron uptake increased secretion of TNF-α in M1-polarized but not in M0 and M2-polarized macrophages and reduced IL-10 in M2-polarized macrophages. Cytokine secretion did not change in myelin-laden macrophages after exposure to iron. Results are expressed as means ± SEM from three separate experiments. *p<0.05; **p<0.01; ***p<0.005 by Student's t test. B. For measurement of reactive oxygen species (ROS), cells were loaded with 30 µM of oxidant-sensitive DCF-DA dye after exposure to iron and fluorescent emission was measured at 540 nm. The experiment was performed in triplicates and repeated three times. The data are expressed as change in fluorescence/8×10^4^ cells (± SEM). Iron uptake increased generation of ROS secretion in non-phagocytosing macrophages in all polarization states, most prominently in M1 polarization. Myelin-laden macrophages did not respond to iron exposure. *p<0.05; **p<0.005 by Student's t test. C. To visualize ROS generation, macrophages seeded on coverslips were incubated with 30 µM FeCl_3_ for 2 hrs and subsequently loaded with 30 µM of DCF-DA. Images were acquired at timed intervals (representative images at 10 min are shown). The results recapitulate ROS quantification in (B): ROS was increased in iron-laden MDMs [d–f] compared to naïve [a–c] or myelin-laden MDMs [g–i]. In contrast, labeling for the M2 polarization marker CD206, revealed decreased expression of CD206 in macrophages exposed to iron [dd–ff] and increased expression in myelin-laden macrophages [gg–ii].

To assess the functional consequences of iron uptake, macrophages were loaded with iron or with iron and myelin and release of TNF-α and IL-10 were measured by ELISA (Fig. 4A) and generation of reactive oxygen species (ROS) was measured with the fluorescent indicator DCF-DA (Fig. 4B). Moreover, formation of ROS and expression of CD206 was visualized on coverslips in macrophages exposed to iron or iron plus myelin (Fig. 4B, C).

Iron induced a dose-dependent release of TNF-α in M1-polarized but not in non-polarized or M2-polarized macrophages. Interestingly, IL-10 release was reduced by iron-uptake in M2 macrophages (Fig. 4A) but not in M1-polarized or non-polarized macrophages (data not shown).. Generation of ROS was significantly enhanced by iron uptake in all three polarization states (Fig. 4B, C). As expected, myelin ingestion prevented all iron-induced changes. Finally, expression of the M2 marker CD206 was reduced in macrophages after iron uptake (Fig. 4C dd–ff) and increased with myelin phagocytosis (Fig. 4C gg–ii).

### Phase hypointense lesions are more prevalent in active relapsing-remitting (RR) than in secondary progressive (SP) MS patients

Next, we examined in MS patients whether iron deposition in white matter MS lesions is associated with increased disease activity. For this, patients with either highly active RR disease or with chronic SP-MS (patient demographics described in table 1) were imaged with ultra-highfield MRI (7 Tesla) gradient-echo/phase sequences and with medium-highfield (3 Tesla) standard sequences (T2WI and FLAIR).

**Figure 5:**
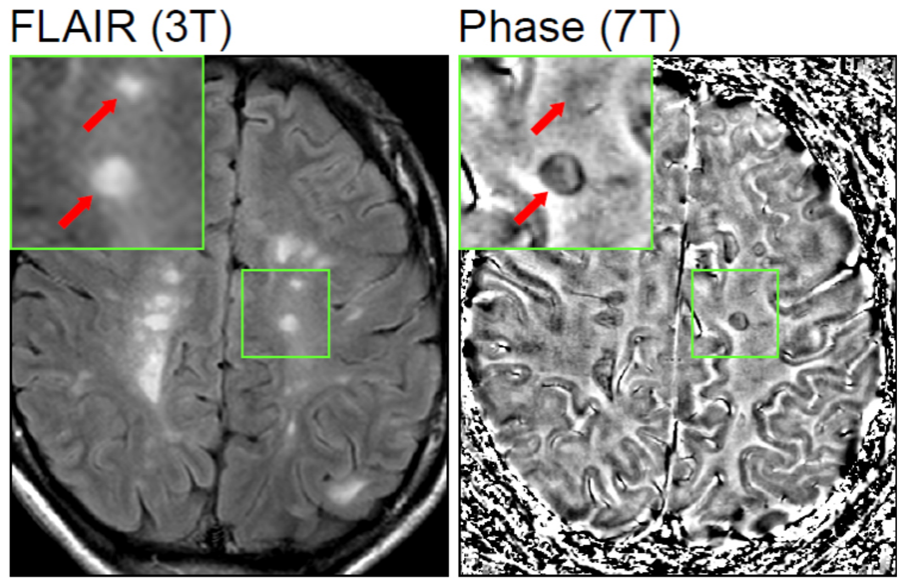
FLAIR (3T) and GRE phase (7T) images of a patient with active relapsing-remitting MS. FLAIR images show numerous white matter MS lesions of which 2 are magnified (inset, red arrows). Phase imaging at 7T phase/GRE reveals a hypointense ring corresponding with one lesion on FLAIR. The other lesion is not visible on 7T GRE (inset, arrows).

**Figure 6:**
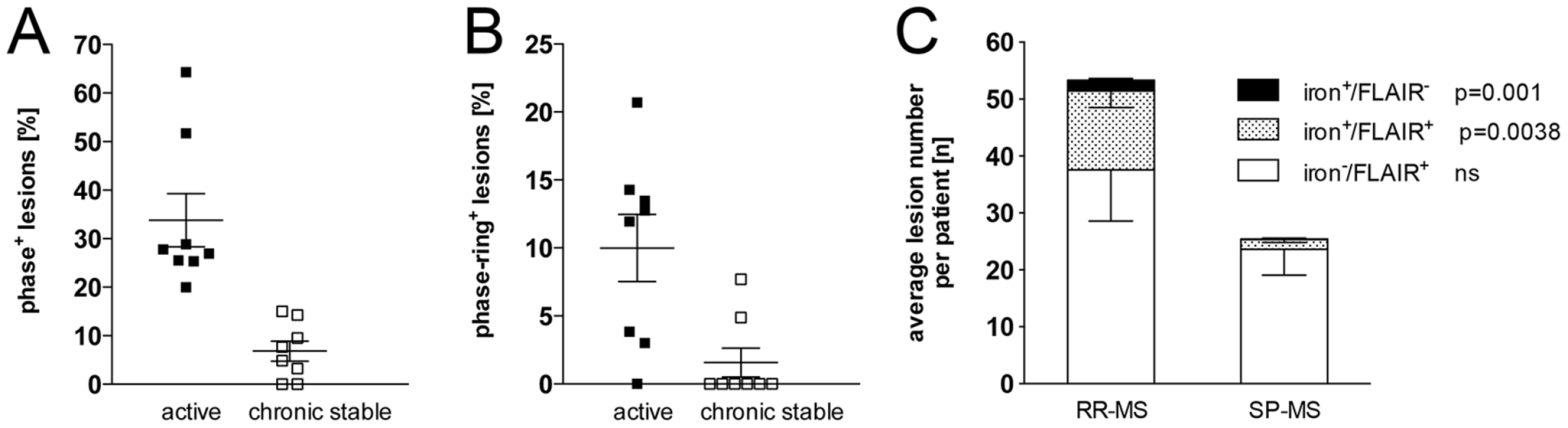
Prevalence of iron-positive lesions in active RR-MS and in SP-MS patients. Patients were imaged with 3T (FLAIR) and 7T (GRE phase) and white matter MS lesions on FLAIR and on GRE phase images were quantified. The percentage of hypointense lesions (all hypointense lesions or lesions with hypointense rim only) that were also visible on corresponding FLAIR images was significantly higher in active RR-MS compared to SP-MS [A, B]. Similarly, the average numbers of phase-positive lesions visible on FLAIR (phase^+^/FLAIR^+^) and phase-positive lesions not visible on FLAIR (phase^+^/FLAIR^−^) was significantly higher in active RR vs. SP patients. In contrast, white matter MS lesions on FLAIR that were phase isointense (phase^−^/FLAIR^+^) did not differ significantly between patient groups [C]. (n = 8 patients/group; P values: (A) p = 0.009; (B) p = 0.0136. Data are expressed as means ± SEM).

In the active, relapsing-remitting group, we identified a total of 101 phase-hypointense lesions that were also present on corresponding 3T FLAIR images (Fig. 5, table 1). An additional 13 phase hypointense lesions were identified that were not visible on FLAIR or T2WI (table 1; Fig. 6C). In the SP-MS group, we found a total of 15 phase hypointense/FLAIR-positive lesions and 1 phase-positive lesion not visible on FLAIR/T2WI (table 1; Fig. 6C). The majority of phase-positive lesions displayed uniformly hypointense signal or hypointense phase rings as previously described.

The percentage of all phase hypointense lesions, visible on corresponding FLAIR images, was significantly higher in active RR-MS compared to SP-MS (33.8% vs. 6.8%; Fig. 6A, C). Similarly, the percentage of lesions with hypointense phase rim was significantly increased in active vs. chronic MS (10.0% vs. 1.6% respectively; Fig. 6B). In contrast, the average numbers of white matter MS lesions visible on FLAIR that were not phase-positive was similar in both groups (Fig. 6C).

## Discussion

With the present study, we provide evidence that iron accumulates within a subgroup of chronically demyelinated lesions but not in actively demyelinating lesions. The iron-containing lesions typically displayed no or minimal active demyelination at the lesion edge and can therefore be classified as non- or slowly-expanding according to Lassmann [18]. Within chronically demyelinated lesions, iron was present in microglia and macrophages predominantly at the lesion edge. Macrophages localized at the lesion center containing condensed myelin debris, were typically iron-negative.

Similarly, the densely packed, myelin-laden (foamy) macrophages in actively demyelinating lesions were uniformly iron-negative. Thus, based on iron content, two macrophage populations can be distinguished in MS lesions: iron-positive macrophages that do not contain myelin and myelin-laden, iron-negative macrophages.

These findings were replicated in human monocyte-derived macrophage (MDM) cultures. Iron was readily taken up by naïve macrophages; however, uptake was significantly reduced in myelin-containing macrophages. Moreover, active myelin phagocytosis led to the depletion of previously ingested iron from macrophages suggesting that myelin downregulates intracellular iron retention in macrophages. Since ingestion of polystyrene microspheres beads did not prevent subsequent iron uptake, myelin might affect iron homeostasis through a specific, possibly lipid-induced process rather than through phagocytic overload.

Secondly, we demonstrated that iron-positive and iron-negative macrophages assume functionally contrasting activation states. Depending on environmental cues, macrophages can change their functional state and give rise to different cell populations with distinct phenotypes and effector functions [19], [20]. The two opposing extremes of a spectrum of activation states consist on one hand of “classically” activated or M1-polarized macrophages with enhanced microbicidal capacity due to secretion of high levels of proinflammatory cytokines and toxic intermediates. On the other side, “alternatively” activated or M2-polarized macrophages have high scavenging capacity, dampen the proinflammatory response and are essential for late phase tissue repair [21], [22]. Although classification of macrophages into M1 and M2 constitutes a simplification of likely much more complex transitional states of macrophage activation, it nevertheless provides a useful working scheme [23]. It is well documented that iron is retained by macrophages during inflammation. Since iron is essential for many biochemical activities, iron retention serves to reduce the extracellular availability of iron and thus to limit pathogen growth [24]. Iron handling by macrophages differs substantially in different polarization states with M1 polarization leading to intracellular iron sequestration and M2 polarization leading to enhanced iron release and low intracellular iron [25], [26].

Consistent with these observations, in MS lesions, iron-positive macrophages expressed markers of M1 polarization such as iNOS while iron-negative, foamy macrophages expressed CD206 and arginase-1, indicative of M2 polarization. Similarly, in our *in vitro* experiments, iron was preferentially ingested by M1-polarized macrophages and iron uptake itself promoted enhancement of the M1 phenotype as indicated by increased production of ROS and TNF-α and by decrease in the anti-inflammatory cytokine IL-10. Myelin ingestion inhibited subsequent iron uptake, therefore preventing iron-induced M1 polarization. We speculate that the results in our ^55^Fe uptake experiments underestimate the blocking effect of myelin on iron uptake (Fig. 3). Only 76% (M1) and 87% (M2) of macrophages were myelin-laden after exposure to myelin (data not shown); thus, substantial numbers of non-myelin laden macrophages were present in myelin-exposed cultures whose iron ingestion was not affected.

Overall, our results further substantiate the dichotomy between iron-positive and myelin-positive macrophages: iron-uptake in macrophages promotes a pro-inflammatory, neurotoxic phenotype while myelin-phagocytosis reduces iron uptake/content and thereby prevents iron-induced M1 activation.

The present report is the first to examine the function of iron-loaded macrophages within MS lesion. Our findings might be particularly important with regard to chronic, macrophage-driven inflammation. A recent study on chronic venous leg ulcers has shed light on the role of macrophage iron in perpetuating inflammation [27]. In this condition, macrophages phagocytose locally extravasated erythrocytes leading to accumulation of intracellular iron. This iron overload induces an unrestrained proinflammatory M1 phenotype in a subpopulation of macrophages that fails to convert to an anti-inflammatory M2 phenotype as it occurs during normal wound healing [28]. This persistence of proinflammatory macrophages results in chronic inflammation with continuing tissue breakdown and impaired wound healing [27]. We speculate that in analogy to chronic venous ulcers, iron accumulation within macrophages at the edge of chronic active MS lesions might propagate chronic, neurotoxic M1-type inflammation.

With regard to the effect of myelin on macrophage function, our findings are in line with previous work by Boven *et al.* [29] who found that foamy macrophages in active MS lesions consistently expressed anti-inflammatory markers. Moreover, prolonged incubation of cultured human macrophages with myelin resulted in expression of anti-inflammatory mediators and in a muted response to pro-inflammatory stimuli and the authors concluded that myelin-laden macrophages within MS lesions may contribute to resolving inflammation and to promoting lesion repair [29].

The geographical distribution of iron-positive macrophages within MS lesions and the specific iron-induced phenotype delineated above provides a basis to interpret iron-sensitive phase imaging in MS patients. The pattern of iron distribution seen in histological sections of MS lesions is consistent with the characteristic phase rings present in a subset of white matter MS lesions on phase imaging [5], [9]. Thus, phase rings within lesions on MRI likely represent iron-positive, M1-polarized microglia/macrophages at the edge of slowly or non-expanding lesions. However, other factors than iron may influence phase imaging signal. It is possible, that effects from myelin contribute to the observe phase patterns and future studies are necessary to evaluate this effect. Similarly, the histological equivalent of nodular phase lesions that have been consistently observed in this and other MS imaging studies [9], [30], [31] is less apparent. The two recently published papers that correlated ultra-highfield imaging of *post mortem* tissue with histochemical detection of iron [7], [31], made no reference to homogenously iron-positive white matter MS lesions. In our histological sample of 53 lesions, we did not find uniformly iron-rich lesions, although numerous lesions contained various degrees of iron-positive cells within their center.

Our study also confirms previous reports that have identified additional sources of iron within the CNS, i.e. iron-rich oligodendrocytes in myelinated white matter and iron-containing perivascular hemosiderin depositions within and outside of lesions [7], [16]. The presence of iron within white matter oligodendrocytes manifests as diffuse, faint MRI phase changes outside of white matter MS lesions and hemosiderin depositions appear on phase images as patchy foci of decreased phase signal [7]. Both can be easily distinguished on MRI from phase rings and homogenously hypointense (nodular) lesions. A major limitation of this study is that we have not imaged the autoptic brain tissue prior to immunohistochemical analysis. A direct correlation between phase signal and immunohistochemical findings was therefore not possible. However, a number of prior studies including our own, have made these correlations [6], [7], [31].

A major finding of the present study is that phase hypointense lesions (both ring and nodular phase lesions) were significantly more prevalent in patients with high inflammatory activity (relapse and/or Gd-enhancement within the last 9 months) than in patients with secondary progressive disease (no relapses, stable white matter MS lesion load, no Gd-enhancement).

These findings are consistent with a study that examined the lesion ages at which phase hypointense signal (and tissue susceptibility) becomes most prominent. The authors found that phase hypointensity increased rapidly within the first 0.5 years and peaked at 0.5–3 years (Yi Wang, manuscript submitted).

The association of phase hypointense signal with active relapsing-remitting MS and with an early to medium lesion age further substantiates our hypothesis that hypointense phase signal represents a distinctive type of inflammatory activity.

Combined with previous correlative imaging/histopathology studies [7], [31] and our own histological and functional data presented here, these data suggest that phase lesions, at least in the form of hypointense rims, are indicative of iron-loaded, M1 activated microglia/macrophages.

By distinguishing iron-positive, M1-polarized and thus potentially neurotoxic macrophages from iron-negative, myelin-laden, M2-polarized and thus anti-inflammatory macrophages, phase imaging highlights a specific aspect of inflammatory activity in MS patients. Thus, phase imaging provides additional information to gadolinium enhanced MRI and to experimental imaging modalities of activated microglia/macrophages through PET imaging [2] or through trace infiltration with USPIO-loaded monocytes [3], which are not known to distinguish between different polarization states of microglia/macrophages.

In order to confirm phase signal in white matter MS lesions as a marker for proinflammatory microglia/macrophages and to validate its clinical relevance, larger cross-sectional and longitudinal studies are necessary. In ongoing studies we are evaluating whether phase signal is as a predictor of tissue damage within lesions and are characterizing the relationship between gadolinium enhancement and iron deposition (manuscript in preparation).

Although 7T imaging has intrinsic advantages over low- and medium-field MRI, iron-sensitive GRE sequences can be performed at lower field strengths [4], [8] and thus can be broadly applied to the clinical setting. Ultimately, iron-sensitive imaging might become a standard MRI technique that provides relevant information on the inflammatory status of MS patients and sheds light onto underlying disease processes.
